# A Discussion of Vaginal Hysterectomy with Observations of Eleven Cases with One Death1Read at the Annual Meeting of the American Association of Obstetricians and Gynecologists, September, 1890.

**Published:** 1891-02

**Authors:** Charles A. L. Reed

**Affiliations:** Cincinnati; Professor of Gynecology and Abdominal Surgery in the Cincinnati College of Medicine and Surgery; Professor of Gynecology at the Polyclinic; 311 Elm Street


### A DISCUSSION OF VAGINAL HYSTERECTOMY WITH OBSERVATIONS ON ELEVEN CASES WITH ONE DEATH.[1]

By CHARLES A. L. REED, M. D., Cincinnati.

Professor of Gynecology and Abdominal Surgery in the Cincinnati College of Medicine and Surgery; Professor of Gynecology at the Polyclinic.

Dr. Charles Egerton Jennings, in his recent masterly work on cancer,[2] assumes the position, in cases in which that malady involves the womb, that partial excision of the organ should be practised when the disease is recent and "localized in the lower part of the cervix;" but that "if the disease has already extended so far that normal mobility of the uterus is diminished, the mere excision of the intra-vaginal, or even the supra-vaginal portion of the cervix will probably be quite inadequate to prevent the recurrence of the disease. Therefore, the anticipated recurrence should be forestalled by excision of the entire uterus."

The foregoing is such a complete summary of the fallacious doctrines of treatment — all the more dangerous because they contain some truth — that are happily only occasionally met with nowadays, but that sometimes come with such a stamp of authority — as in the present instance — that I take the quotation as a text for the further discussion of the important subject of vaginal hysterectomy. It would seem that such a discussion was not needed in the midst of the voluminous literature on the subject, which has come like an avalanche upon the profession within the last few years; but careful attention to that literature will reveal the fact that surgical treatment of cancer of the womb is not yet fixed upon definite lines. It falls to the experience of all of us to almost daily

---

1. Read at the Annual Meeting of the American Association of Obstetricians and Gynecologists, September, 1890.

2. Cancer and its Complications. By Charles Egerton Jennings, F. R. C. S., etc., London, 1889.

encounter cases of this malady that have either neglected themselves or have been neglected by their physicians, or have been flatly advised by the latter against any operative interference whatever. So long as this state of affairs exists, just so long should writers continue to emphasize the fact that cancer of the womb is a disease capable of cure by extirpation. It falls to the experience of most of us, also, to encounter vacillating opinions among operators as to the proper course to be pursued when these cases do become applicants for surgical relief. So long as this state of affairs continues, and so long as it finds warrant for its continuance in expressions such as I have quoted, just so long should the controversy be kept open.

In approaching a discussion of this question we find just two propositions which are accepted by the profession as axiomatic, viz.: (1) cancer of the womb, if not treated, is uniformly fatal: and (2) treatment, to be effective, must involve removal of all the cancerous tissue. The pathological conditions antecedent to these conclusions have been held open to debate, but, save Mr. Buntlin, I know of no reputable authority that today contends for the primary constitutional origin of cancer. The theory of Waldeyer, as subsequently confirmed by Tait, viz., that cancer is always primarily of epithelial, and consequently of local, origin is accepted with practical unanimity. If we use the word epithelium as the generic term embracing endothelium of blind follicles within its meaning, I cannot discover that latter-day observations have in the least disturbed this well-demonstrated doctrine. If, now, cancer is thus always primarily local in its origin, and if, as has just been declared, it is uniformly fatal if not treated, it follows that the disease, essentially progressive in character, must advance either by the continuous invasion of adjacent normal tissue or by migration of cell-elements either through the lymphatic or hemic circulations. The histological arrangement of strictly cancerous growths prevents the easy invasion of the hemic channels, and the lymphatics, present in the uterus in large numbers, are generally so dormant in the non-gravid womb, that they do not serve as avenues through which metastatic changes are effected ; it follows, therefore, that the advancement of these growths depends upon the progressive invasion of the normal matrix. This manifestation of malignancy, in turn, depends upon rapid endogenous cell-proliferation—a power inherent in one as well as in many of these constituent cells ; it again follows, therefore, that an attempt at removal which will leave a single one of these cells will, by that omission, leave a nidus for the invariable redevelopment of the disease. This train of thought brings us forcibly to the second conclusion, already announced, viz., that the only way to cure cancer effectually is to remove absolutely all its elements.

Thus far I feel that I have encountered no serious dispute, but the next question that arises is, How are we to make most sure of the removal of all the disease? This brings me at once to my controversy with Dr. Jennings, and with those who, like him, affect a course of conduct which they choose to call conservative. I believe in that conservatism which conserves human life, but I do not believe that Dr. Jennings's conservatism best subserves this important end.

A fairly industrious research among the most recent writers on the pathology of uterine cancer confirms my own limited observation that, cases of epithelioma aside, malignant disease of this organ has its initial lesion in the endothelium of the submucous follicles, and that the extension of the disease is by the progressive invasion of the submucous tissues. This is one of the most important facts to be taken into consideration when we attempt to judge of the extent of the disease before operation; for it will be seen at a glance that while we may remove a segment of the cervix by means of the scissors, and while we may scrape the canal with the curette to secure aberrant epithelium for microscopical examination, we will yet be utterly unable to determine the farthest invasion of the disease, for the reason that we cannot by those means reach the deeper tissues that are concerned in the early metamorphosis. I cannot in the least agree with Dr. Jennings that the extent of the disease can be accurately determined at the time of the operation of amputation, and I doubt if he can get those who have had the most to do with cancerous uteri to agree with him in this regard. Our most careful pathologists inform us that it is extremely difficult to make out the marginal lines between the areas of cancerous infection and normal tissue; and if it be difficult to settle this important question by means of powerful lenses in the leisure of the laboratory, how impossible must it be to arrive at an accurate conclusion on the same subject by means of the naked eye in the necessary hurry of the operating-room? My first objection to the operation of high amputation, even in cases of early cancer apparently localized in the lower part of the cervix, is that, in consequence of the method by which the disease extends—a method recognized by

Dr. Jennings himself — we cannot know that all the diseased tissue is removed, and that the operation is, for this reason, unsurgical.

Dr. Jennings, in speaking of high amputation of the cervix, alludes to it as an operation "which can be performed without opening the peritoneal cavity and with very slight risk to life." I am bound to admit that this view is very generally accepted, but I must, at the same time, urge that it is but another instance of the popularity of error with the profession. That the operation can be done without opening the peritoneal cavity is conceded, but that it is frequently not done without opening the peritoneum, is likewise true. This fact, however, has no surgical significance one way or the other. That the operation offers but "slight risk to life," as compared with vaginal hysterectomy, is a declaration the accuracy of which I beg to challenge. In doing so, I fully concur with that distinguished English operator, Professor Sinclair, of Manchester, who recently stated[1] "that, although vaginal hysterectomy was called 'major,' it was, perhaps, less dangerous than some of the so-called 'minor' operations"; and with Dr. Cullingworth, who, on the same occasion, stated that, after careful study of vaginal hysterectomy in Germany and of high amputation in England, he had found that the mortality of the former was five per cent., while that of the latter was seven per cent., giving a difference of two per cent. in favor of the so-called "major" operation. I am not one of those who accept distinguished opinions, however authoritative, nor favorable statistics however accurate and conclusive, unless the question which they apparently settle is susceptible of rational demonstration. When we remember the almost utter impossibility of keeping the field of operation in high amputation free from subsequent infection, even in ordinary cases, we realize one of its dangers. One of the two cases that I have lost during the last year from sepsis was one of high amputation of the cervix for non-malignant conditions, and one in which antiseptic details were carefully observed. If, then, the danger is so pronounced in non-malignant cases, how much more marked must it be in cases in which is to be added to the danger of infection from decomposing secretions the greater danger of contamination by cancer cells, either from the segment removed or from the remaining portions of the womb. I speak of this as the greater danger, and I feel warranted in doing so by the reflection that by the operation both the systemic circula-

1. Lancet, May 17, 1890.

tions—hemic and lymphatic—are opened, and that through the portals thus opened the malignant elements may gain easy access, thence to course their mischievous ways through

"—the natural gates and alleys of the body."

But infection, whether septic or malignant aside, in matter of technique the operation of high amputation is not without its difficulties. The literature of the operation is marred with the record of more than one death from uncontrolled hemorrhage. The certainty of hemostasis is much greater, particularly with forcipressure, in total extirpation than in high amputation. In one of my cases I found, after dividing the mucous membrane around the cervix, that the carcinomatous invasion had extended beyond the uterus. Acting under a mistaken idea, I endeavored to conclude the operation by doing a high amputation, but I was forced to remove the entire organ to bring the bleeding tissues under control. My second objection to high amputation is, that it is a more difficult and a more dangerous operation than vaginal hysterectomy.

But if it were possible to know that all the diseased tissue had been removed, and if it were true that the operation were easy and safe, I would still be deterred from performing high amputation for a reason that I have not seen mentioned, but which I now earnestly commend to the attention of the Association. It has doubtless fallen to the Fellows, as it has frequently to me, to be called upon to dilate the stump of a cervix, the lower portion of which has previously been excised for the relief of either dysmenorrhea or sterility, or both. It requires no histologist to determine what many a histologist has determined, that the contraction and inelasticity of the stump are due to the presence of a small ring of cicatricial tissue. This ring, in magnitude and consequent inelasticity, is proportionate to the mutilation which it has repaired. As the maximum of mutilation is realized in high amputation, it follows that the maximum of scar tissue is deposited in these cases; so that, even in instances of non-recurrence of the malignant disease, the base of the remaining portion of the uterus, while pervious, is yet hard and inelastic. In other cases, in which the disease has been simply repressed for a time, but in which with characteristic insidiousness it has recurred, the uterine canal may remain patulous. In either event pregnancy is liable to occur. At the recent meeting of the American Medical Association this fact was urged as a reason for high amputation. It would have been entirely easy for the eloquent speaker on that occasion, himself one of the distinguished operators of America, to have availed himself of the particulars of the following case, which I quoted in my rejoinder to his remarks at the time:

Mrs. H., æt. thirty-four, wife of a clergyman; was never robust and never pregnant; was treated during Spring and Summer of 1888 for excessive menstruation. In July the bleeding became so great and so persistent that her physician examined her in August and found bleeding nodules in the cervix. Treatment was of no avail, so that in October she was sent with a letter to Dr. ——. Epithelioma was diagnosticated, and an operation advised. The consultant came to her home to do vaginal hysterectomy, but instead did high amputation on November 13, 1888. Present and assisting were the writer and three physicians of the town, including the patient's attending physician.

Now, right here, comes in a curious fact in point of diagnosis and treatment. Observe that this operation was done November 13th. In the letter which the patient carried to the consulting, now operating, gynecologist, attention was called to the fact that somewhere in September, 1888, this woman suddenly and unexpectedly, and without regard to treatment, ceased to bleed, and asked if this did not throw some doubt on the diagnosis of cancer. Thus, for a space of at least six or seven weeks a woman with a cancer of the cervix did not bleed until the moment of operation.

She was instructed to visit the operator in ten or eleven weeks, which she did. Not having menstruated or bled one drop since the operation, the question was raised, Could she possibly be pregnant? This proved to be the case, and she had splendid health up to her labor, which began on June 20, 1889, under the care of her family physician.

Palpation showed undoubted uterine contractions. Digital examination revealed—*a blank!*—no os, no cervix, nothing, save a thick mass of cicatricial tissue over the lower end of the womb, and a vertex presentation. At the end of twenty-four hours there were still no signs of an opening. The doctor incised the point that seemed to bulge the most for an inch and a half; slight bleeding. This was of no avail, and after twelve hours he enlarged this incision. He now had a consultation, and between them they incised several times at the point of this gristle that appeared to be on the greatest strain. At last they made the passage large enough for the head to pass. Forceps were then applied, and labor completed June 24, 1889, at 9.15 A. M. The patient had already one chill, soon followed by a temperature that went to 106°; she had another immediately after delivery. Septic peritonitis followed by death, June 27th, 9 P. M. Child died long before—several hours before delivery.

From the foregoing graphic particulars, which I have from one of the most accomplished practitioners in the State of Indiana, and whose narrative I have modified only by unimportant omissions in the interest of brevity, furnishes us with food for reflection. It would be possible to take up the question of diagnosis of cancer ; it would be feasible to raise questions as to the propriety of treatment, taking into consideration the alleged character of the disease and the pregnancy which, computed from the delivery at term, must have been of nearly seven weeks' standing at the time of operation ; and it would be entirely just to enter the strongest possible protests against the failure to do a Cesarean section at the time of delivery. I prefer, however, to pass all these questions by for the sake of giving emphasis to one point, viz., death occurred in this case as the result of conditions at the base of the uterus, consequent upon, and induced by, the operation of high amputation of the cervix. If this were an isolated case it would have less significance. Goodell has had occasion during last year to do Cesarean section on a woman with a cancerous cervix, but whether recurrent after high amputation or not I am not prepared to state. Spencer Wells, Bischoff, and Thomas are reported to have had similar cases. My third objection, therefore, to supra-vaginal excision of the cervix is the fact that pregnancy may ensue, and that whether the disease returns or not, the conditions at the outlet of the uterus are such as make delivery by abdominal section the safest means of saving either the mother or the child.

Dr. Jennings, in the passages which I have quoted, advises total extirpation in those cases in which the disease has "already extended so far that the normal mobility of the uterus is diminished." This is my most serious cause for complaint against the distinguished author, for it is precisely this kind of advice that is responsible for all of the mischief following vaginal hysterectomy for cancer. The loss of normal mobility of the uterus under these circumstances means extension of the disease beyond that organ, and when such extension has taken place no operation compatible with primary recovery can eradicate the mischief. It was the failure to appreciate this latter fact, and the obstinate persistence of early operators to subject all recent cases to partial excision and to relegate only advanced ones with lessened mobility to total extirpation, that was chiefly to blame for the bad statistics of the latter operation in the years now happily past. Recent successes by the abdomino-vaginal method show that the primary mortality of 67.2

per cent. on a basis of 119 cases, operated upon by that method and reported, must be attributed as much to delay in undertaking the operation as to any defect in the technique. The statistics of Hoffmeier and Schroeder relative to the ultimate results of the two operations, on a basis of 136 high amputations and seventy-four vaginal hysterectomies, showing 43.3 per cent. of the high amputations well and all the total extirpations dead after four years, are absolutely worthless for the same reason. If Dr. Jennings's teach ings are to be taken as an index of English practice today, we at once have the explanation of the fact stated by Mr. Lewers, in a recent discussion before the Obstetrical Society of London, that but thirty per cent. of his cases were without recurrence after two years. In my own experience, based upon eleven cases, with but one primary death, full reports of which, with names of attending physicians, will be found in the transactions of the Ohio State Medical Society for this year, I have had some occasion to study the disastrous effects of delay in operating, but a delay for which I hasten to declare that I was in no instance responsible. My one strictly primary death was in a patient in whom the disease had existed for at least thirteen months, and who was almost exsanguine at the time of the operation. That which we call cachexia in these cases is really but a form of anemia induced by the frequent and persistent bleeding, which lessens the powers of resistance and makes the patients the easy victims of surgical shock. It was from this condition that the patient to whom I have just alluded died, although the operation was a short and easy one, and the disease was yet clearly limited to the uterus.

I have had two cases in which recurrence took place after total extirpation, and with what appeared to be complete removal of the disease. The first of these, a case of medullary cancer of the endometrium of thirteen months' standing, was operated upon January 11, 1888, and was reported to the Obstetrical Society of Cincinnati[1] the following month. The condition of the specimen after the curetting preliminary to the operation, was such that distinguished members of that Society expressed their doubts as to the existence of the disease, and significantly expressed the opinion that the case was one in which the disease would not recur. Evidences of the return of the disease were, however, manifest after twenty months. She died at Randolph, Maine, August 28, 1890, having lived to within about three months of three years after the

1. American Journal of Obstetrics.

operation. I quote briefly from the report of the very thorough autopsy made by Drs. Sawyer and Pitt, August 30th, and kindly communicated to me by Dr. Pitt. The examination was evidently very carefully made, and involved all the abdominal and pelvic organs. I shall, however, quote only such portions as relate directly to the point:

*Omentum* fatty; a mass was found projecting three-fourths of an inch, one-fourth of an inch in diameter, white, translucent, like jelly, but harder in consistence. Another was a small red mass like a polyp, the size of a filbert. There were also calcareous patches.

Bowels were thickened irregularly throughout, in many places three times their normal thickness. No ulcerations, but the whole mucous membrane so congested that the blood seemed exuding from the whole surface. The secum was adherent to a mass in the right iliac region, to which was also adherent the right broad ligament. There did not seem to be any ovary there, nor did it appear to have been removed, so must have degenerated. [Neither ovary was removed at the operation.—REED.] The whole mass was hardened and attached to the deeper tissue, covering a space of three and a half by two inches. The inner surface of the cecum was normal, but in place of the appendix ceci was a hole from which depended a mass of red granulations through the opening between the vagina and peritoneal cavity. This opening into the vagina was perfectly healed and smooth, with no granulations upon it, and formed a ring three-quarters of an inch in diameter. The mass of granulations projecting through this ring was the size of a small egg, dark-red in color, soft and friable, with so little connective tissue that it broke down at touch into a bloody mass.

Aside from a perforation of the colon at its juncture with the sigmoid flexture, a pus cavity in the right kidney, which was atrophied, and a calcareous left ovary, no other essential pathological conditions are recorded in this model report from the pen of Dr. Pitt. Specimens of the granular tissue were preserved, but I have not as yet had the opportunity of seeing them. They will be subjected to microscopical examination and a report will be added as an appendix to this paper. From the microscopic appearances so graphically recorded, there can be no doubt that the case was one of "secondary medullary cancer of the peritoneum," a condition recently described by Mr. Fenwick in his *Clinical Lectures on Obscure Diseases of the Abdomen* (London, 1889). This probability is increased by the statement of Mr. Fenwick, that 80 per cent. of the cases observed by him followed primary manifestations in either the uterus or its appendages, the kidneys, or the bones.

My second case of recurrence was carcinomatous in character, and had been observed to exist for seven months, although the history of menorrhagia, verging into metrorrhagia, extended nearly a year farther back. The disease in this instance recurred at the site of removal, although the patient had an interval of good health for six months. In another case of nine months' standing, the disease was found to have invaded the broad ligaments at the time of operation. Of my eleven cases, the average previous duration of the disease was about eight months. Of those now living and well, the average previous duration was but a fraction over five months. One is now living within two months of three years after the operation, another after two years and five months, while the most recent case is of six months' standing.

The progressive advancement of this operation during the last ten years is a source of gratification to its friends. The various collections of cases indicate a diminishing primary mortality :

| Year. | Name. | Cases. | Mortality. |
|---|---|---|---|
| 1881 | Olshausen | 40 | 29 per cent. |
| 1883 | Sänger | 133 | 26.6 " |
| 1884 | Engstroem | 157 | 29 " |
| 1886 | Hegar and Kaltenbach | 257 | 23 " |
| 1890 | Martin (F. H.) | 134 (American) | 14 " |

When it is remembered that these are collective statistics, and represent the early experience of many operators, they will be construed as vastly more favorable than they seem. The recent record of individual operators is very satisfactory. The best so far achieved, I believe, is by Kaltenbach, who, at the recent Hegar celebration, reported sixty-two consecutive cases with but two deaths, giving a mortality of but 3.2 per cent. We shall realize precisely the same results when our experience grows, and when the general profession—into whose hands these cases first come—realize that it is their duty to make an early diagnosis and act upon the clearly-established principle that every woman who has cancer of the uterus, and in whom the disease has not lessened the mobility of the organ, should be subjected to the operation of vaginal hysterectomy.

I believe these considerations justify me in the statement that the delay which is called conservatism, and which is practically advised in the paragraph which I have quoted from Dr. Jennings, is radically wrong. The prospect of improving this operation must come by subjecting early cases to total extirpation, and in declining any operation whatever to those in which the disease has gone beyond the uterus. It is out of the question to restore to health that woman whose womb has lost its normal mobility by the advancement of cancerous disease. As well might one try to make a vigorous man out of the charred remains of the victim of electrocution, or to reconstruct a breathing Cleopatra out of a shriveled mummy from the catacombs of Egypt.

311 Elm Street.

